# The anatomy of a comprehensive constrained, restrained refinement program for the modern computing environment – *Olex2* dissected

**DOI:** 10.1107/S2053273314022207

**Published:** 2015-01-01

**Authors:** Luc J. Bourhis, Oleg V. Dolomanov, Richard J. Gildea, Judith A. K. Howard, Horst Puschmann

**Affiliations:** a Bruker AXS–SAS, 4 Allée Lorentz, 77447 Marne-la-Vallée cedex 2, France; b OlexSys Ltd, Department of Chemistry, Durham University, South Road, Durham, DH1 3LE, England; c Diamond Light Source Ltd, Diamond House, Harwell Oxford, Didcot, Oxfordshire, OX11 0DE, England; d Department of Chemistry, Durham University, South Road, Durham, DH1 3LE, England

**Keywords:** small molecules, refinement, constraints, restraints, least squares, *Olex2*

## Abstract

This paper describes the mathematical basis for *olex2.refine*, the new refinement engine which is integrated within the *Olex2* program. Precise and clear equations are provided for every computation performed by this engine, including structure factors and their derivatives, constraints, restraints and twinning; a general overview is also given of the different components of the engine and their relation to each other. A framework for adding multiple general constraints with dependencies on common physical parameters is described. Several new restraints on atomic displacement parameters are also presented.

## Abstract 2

An in-depth presentation is given of *olex2.refine*, the new refinement engine integrated in the *Olex2* program.

## 1. Introduction   

During the last four decades, small-molecule crystallographers have used a myriad of stand-alone routines and various comprehensive software packages, most notably those written by Sheldrick (1997 ▶, 2008 ▶) and that designed by Bob Carruthers and John Rollett, then maintained and enhanced by David Watkin (Betteridge *et al.*, 2003 ▶). These latter two packages have dominated the citations in all publications containing crystal structure analyses for many years now.

Therefore it might be claimed that the analysis of single-crystal X-ray (and neutron) diffraction data has reached a certain level of maturity and that there is no need for further program development. Nonetheless, there is still considerable activity in this area by various groups around the world and there has been a new release, *SHELX2013*, from Sheldrick recently. A retro-fit of new ideas to these older programs is not always possible, except perhaps by the authors themselves.

When we first embarked on the project herein reported^1^ we were clear about one point – namely that we wanted to create a new and flexible refinement engine, working in a collaborative environment and to be based on trusted and mature pre-existing code. This new refinement engine would be open source and therefore available for verification and modification by others. Modern programming paradigms, unknown when the aforementioned packages were created in the 1960s, would form the basis of our development. It is hoped that such an architecture will allow extensions to the code without breaking the program or endangering the underlying functionality. It is this that we describe below in further detail and which now forms the underlying code for *olex2.refine* (http://www.olex2.org).

We decided to base the computational core of *olex2.refine* on a pre-existing project, the Computational Crystallography Toolbox (*cctbx*), that provides the foundation for the macromolecular refinement facilities in the *PHENIX* suite of Adams *et al.* (2010 ▶). We made that choice because it provided the solid and versatile tools (Grosse-Kunstleve & Adams, 2003 ▶) that we needed to develop our project. The most important of them is a comprehensive and robust toolbox to handle crystal symmetries (*sgtbx*) that supports every space group in any setting, even rather unusual settings such as tripled cells. Another key *cctbx* toolbox is the *eltbx*, which is concerned with scattering-factor computations for any atom or ion and any wavelength that could be encountered in practice. The *cctbx* also featured many of the restraints we needed, in the module *cctbx.restraints*.

For our project, we contributed to it a Small-Molecule Toolbox (*smtbx*) which features, in particular, the constraint framework presented in detail in §3 and Appendix *C* [App appc], and the computation of structure factors presented in Appendix *A* [App appa]. We also contributed several new restraints to *cctbx*, restraints which are discussed in §4 and Appendix *D* [App appd]. Finally, both the *cctbx* and the *smtbx* use the Scientific Toolbox (*scitbx*) for arrays, matrices, special functions and other purely mathematical matters. We have contributed a new least-squares toolbox (*lstbx*) to the *scitbx*, which provides flexible tools to deal with generic linear and non-linear problems, with or without an unknown overall scale factor. It implements the method based on normal equations presented in Appendix *B* [App appb].

**Figure 1:**
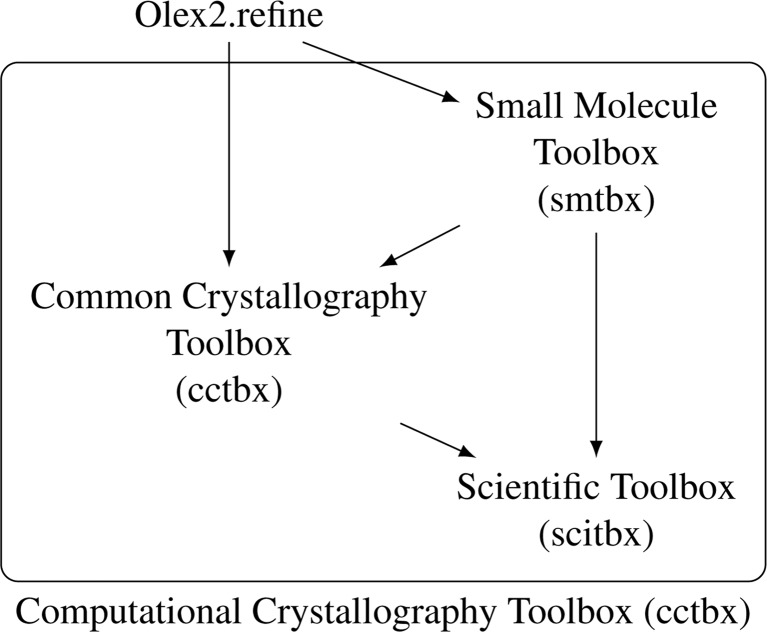
A high-level depiction of the software modules involved in *olex2.refine*: an arrow is drawn from each component pointing towards another component it uses.

The program *Olex2* relies either on *SHELX* or on the *smtbx* to refine structures. If using the latter, *Olex2* converts its internal model of a structure into the objects used by the *smtbx* and the *cctbx* to represent the unit cell, reflections, crystal symmetry, constraints, restraints *etc*. Once the *smtbx* has produced the desired results they are sent back to *Olex2* which converts them to its own representations, so as to perform diverse post-processing and eventually display the results to the user of the program. Fig. 1 ▶ summarizes the dependencies between the various modules and programs that have just been described.

## 2. Least-squares refinement   

A small-molecule structure refinement typically minimizes the weighted least-squares (LS) function
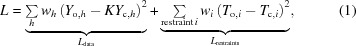
where 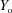 (respectively, 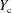) denotes either the measured amplitude 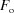 (respectively, the modulus of the calculated complex structure factor 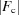) or the measured intensity 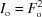 (respectively, the calculated intensity 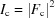), whereas *K* is an overall, unknown scale factor that places 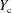 on the same scale as 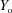. The first sum over *h* runs over a set of *m* non-equivalent reflections that have been observed. Each observation is given an appropriate weight, 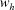, based on the reliability of the measurement. These may be pure statistical weights, 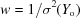, where σ is the estimated standard deviation of 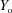, though more complex weighting schemes are usually used. In the most general case, 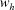 is a function of 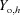, 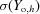, 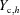 and *h* itself, whereas the most common weighting schemes exhibit only a dependence on the first three.

These X-ray observations can be supplemented with the use of ‘observations of restraint’, as suggested by Waser (1963 ▶), where additional information such as target values for bond lengths, angles *etc*. is included in the minimization. This is the origin of the second term of the sum, where 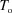 is the target value for our restraint, and 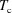 is the value of the target function calculated using the current model (see, for example, Giacovazzo *et al.*, 2011 ▶; Watkin, 2008 ▶). This term 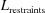 may of course be absent. Conversely, the data term could be absent in a refinement against geometrical terms only [*cf.* the program *DLS-76* by Baerlocher *et al.* (1978 ▶) for example].

In equation (1), the crystallographic parameters [*i.e.* atomic positions, atomic displacement parameters (ADPs) and chemical occupancy in routine refinements] that each component of 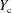 and 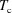 depends upon will be collectively denoted as the vector 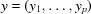 and we can therefore denote those dependencies with the compact notations 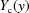 and 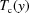. The parameters that are actually refined may be a different set 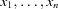, collectively denoted as a vector *x*. We assume that the dependency of *y* upon *x* is known analytically. Since the scale factor *K* is unknown, our problem is the minimization of *L* with respect to all of 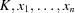. We will denote that dependency as 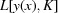, or more tersely when we do not need to remember the crystallographic parameter vector *y*, as 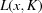. These notations reflect the important fact that we treat the scale factor *K* separately, as will be explained later.

For a small-molecule structure with a high data-to-parameter ratio, such unconstrained minimization as defined by the first term of equation (1), when 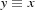, may well be sufficient. However, as the structure becomes larger, or the data-to-parameter ratio worsens, unconstrained minimization may not be well behaved, or result in some questionable parameter values. It has become customary to rely on the use of two techniques to solve such issues:

*Restraints*: by having a non-zero second term in equation (1) – with the use of appropriate weighting of the restraints – the minimization is gently pushed towards giving a chemically sensible and hopefully correct structure.

*Constraints*: by having a parameter vector *x* shorter than *y*, therefore explicitly taking into account exact relationships between some of the parameters 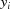.

Whether the refinement uses restraints or constraints or both, at each refinement cycle, we first find the value of *K* that minimizes 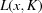 keeping the parameter vector *x* fixed: we will denote it by 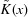, where we use a notation that is explicit in its dependency on the value of the refined parameters at the beginning of the cycle. We then search for the shift vector *s* of the parameter vector *x* that brings 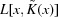 closer to the minimum. It is solution of the so-called normal equations, 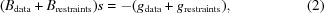
where 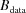 and 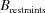 are the so-called normal matrices associated with the two terms sharing the same labels in equation (1), respectively, and where the right-hand side 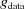 and 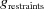 are equivalently associated with those two terms [see the derivation of equation (72) in Appendix *B* [App appb]].

The rest of the paper is organized as follows. §3 deals with the computation of the derivatives with respect to the refined parameters 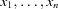 from the derivatives with respect to the crystallographic parameters 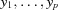. The computation of the latter derivatives is expounded in Appendix *A* [App appa] along with the formulae for the complex structure factors 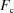. Appendix *C* [App appc] details the computation of the value and derivatives of constrained parameters for each constraint featured by *olex2.refine*. §4 is devoted to the general principles of the computation of 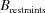 and 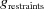. It is completed by Appendix *D* [App appd], which gives the formulae for each restraint featured by *olex2.refine*. §5 deals with the refinement of twinned structures. §6 details the computation of standard uncertainties (s.u.’s), emphasizing the influence of constraints. §7 gives a very quick overview of the output of the results of a refinement. Appendix *B* [App appb] presents the method used to find the optimal scale factor *K*, and then the optimal value of all the other refined parameters.

## 3. Constraints   

### 3.1. Synopsis   

The difference between restraints and constraints may be conceptualized mainly in two manners that we will first illustrate with a simple example: a geometrically constrained acetylenic hydrogen, *X* C—H. The position of the hydrogen must be such that the distance CH is equal to some ideal bond length *d* and such that the angle 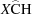 is equal to 180°. Expressed in such an implicit manner, those restrictions could be used to construct restraints by adding to the function to minimize a term *w*_1_(CH − *d*)^2^ + *w*_2_(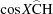 − 1)^2^. By doing so, the number of refined parameters would not be changed but the number of observations would increase by three.

It is, however, traditional to do the opposite, by reducing the number of parameters by three and keeping the number of observations unchanged. This is achieved by using the implicit form of the constraints to express the position of 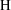 as a function of the positions of the two carbon atoms. Denoting by *x* the triplet of coordinates of the atoms, 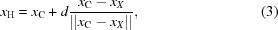
where 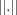 denotes the Euclidean norm. By using this formula, the observable 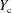 of the fit (either 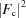 or 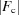) that used to depend on 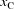, 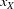 and 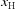 is replaced by a function 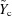 of 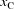 and 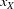 but not of 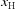. We will call this transformation a reparametrization: 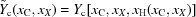 and we will say that 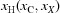 is a reparametrization of 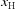, whose arguments are 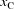 and 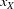.

The derivatives for the remaining variables are obtained with the chain rule 
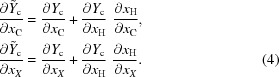
We use the following compact notations for derivatives: for a column vector 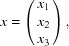


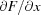
 will always denote the row vector
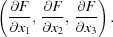
Given another column vector
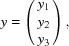


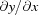
 will always denote the matrix
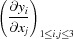
where *i* (respectively, *j*) indexes the rows (respectively, the columns). The identity matrix will be denoted by 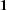.

It should be noted that it is customary to work within the ‘riding’ approximation, 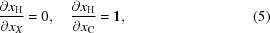
which results in the much simplified chain rule 
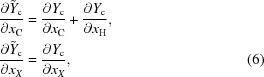
implemented in all refinement programs, including *Olex2*.

It is not always the case that all three coordinates of an atom are removed from the refinement by constraints. For example, an atom in the plane of the mirror 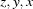 whose matrix reads
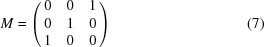
has its coordinates 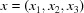 constrained by the relation 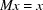, which can be reduced to 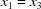 only. The corresponding reparametrization may be written 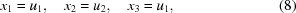
the observable 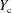 becoming now a function of the vector of newly introduced refinable parameters 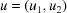. There is a general algorithm implemented in the *cctbx* that for any special position returns a matrix *Z* so that the reparametrization takes the form 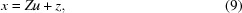
where *Z* is a 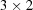 or 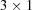 matrix and *z* is a 3-vector. This algorithm first determines the space-group symmetries that leave the site invariant. The resulting system of linear equations, which read 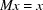 in our example, is then reduced to a triangular form from which the matrix *Z* and the vector *z* are then readily obtained. This algorithm does not therefore try to determine which components of *u*, if any, are also components of *x*. This is why we have presented the reparametrization for our example in this general form instead of keeping 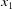 and 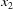 as refinable parameters, which would sound more intuitive in the first place. This matrix *Z* is the matrix of constraints for the position of that atom.

The anisotropic displacement tensor *U* is subject to symmetry constraints as well. In our example, it must satisfy 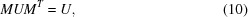
where 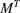 denotes the transpose of the matrix *M* (see *e.g.* Giacovazzo *et al.*, 2011 ▶). Any number of such matrix equations can always be rewritten as a system of equations whose most general form reads 
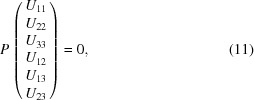
where *P* has 6 columns and 6*n* rows, where *n* is the number of symmetry elements other than the identity involved in the special position [indeed equation (10), being trivial for 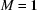, can safely be discarded]. In our example, 
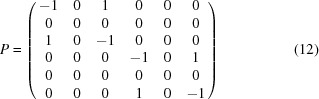
and equation (11) reduces to 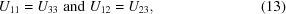
leading to the constraint matrix 
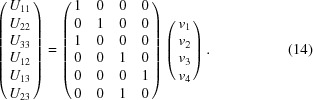
Then one would refine 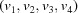 instead of the 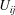. The *cctbx* provides an algorithm that computes this matrix *P* for any special positions, and then reduces it to triangular form in order to determine the constraint matrix for the ADP.

In other cases, a reparametrization will make some crystallographic parameters disappear while introducing new refinable parameters. A typical example is that of a tetrahedral *X*—CH_3_, as the geometrical constraints leave one degree of freedom, a rotation about the axis *X*—C. Thus the reparametrization expresses the coordinates of the hydrogen atoms H_0_, H_1_ and H_2_ as functions of the coordinates of the carbon atoms, and of an angle ϕ modelling that rotation, 
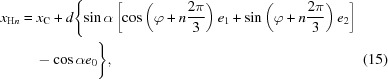
where 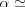 109.5° and 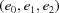 is an orthonormal basis of column vectors with 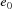 in the direction of the bond 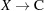. The riding approximation in this case consists of neglecting the derivatives of those base vectors, leading to 
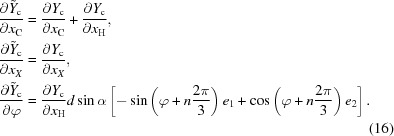
Thus a new derivative with respect to the new refinable parameter ϕ is introduced by this reparametrization.

**Figure 2:**
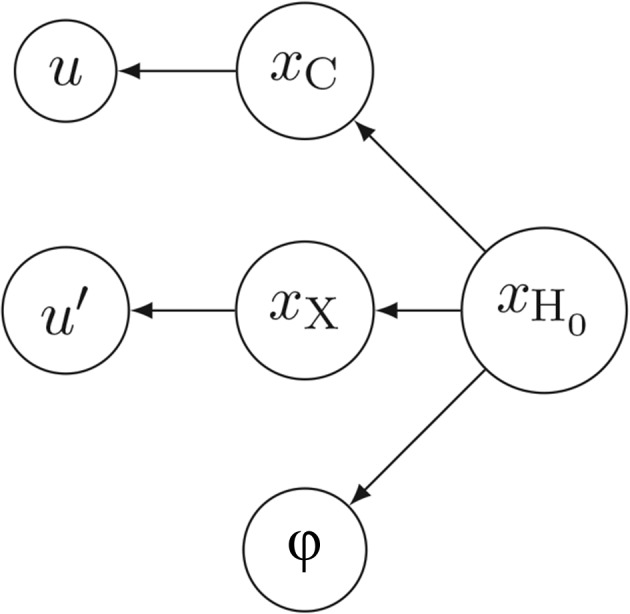
Example of a dependency graph for the chain of reparametrization discussed in §3. Only the part for hydrogen atom H_0_ is shown.

The last important concept is that of the chaining or composition of reparametrizations, that we will illustrate with a combination of the examples above. This example is not particularly common but it is a simple illustration of the concept we want to introduce. In the 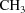 case, the atoms 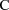 and 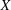 could be on the special position studied in the next-to-last example. One type of disorder could be modelled by first applying the reparametrization (15) and then reparametrizing 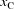 and 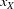 using equation (8), introducing parameters 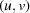 for the former and 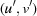 for the latter. The derivatives would then be obtained by the chain rule, *e.g.*

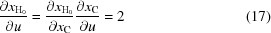
in the riding approximation. This composition of reparametrization may be represented as a graph: each parameter (some of them are triplets of coordinates, others are scalars), 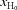, 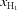, 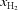, 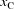, 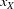, ϕ, *u*, *v*, 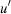 and 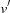, constitutes a node of that graph, whereas edges are drawn from each node to its arguments, *i.e.* the nodes it depends upon, as shown in Fig. 2 ▶. In this example, 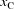 has only one argument, *u*, whereas 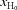 has three arguments, 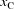, 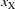 and ϕ. The *smtbx* implements reparametrizations by explicitly building such a graph.

As models become more complex, *e.g.* hydrogen atoms riding on the atoms of a rigid body whose rotation centre is tied to an atom whose coordinates are refined, the reparametrization graph becomes deeper. We decided not to put arbitrary limits on that graph. Indeed, we could have made a closed list of reparametrizations and of reparametrization combinations that our framework would accept but instead we decided to write our code so that it could correctly handle the computation of parameter values and of partial derivatives for arbitrary reparametrizations, combined in arbitrary ways. This framework is therefore open as new types of reparametrizations can be added, without the need to change the basic infrastructure in any way, and without the risk of breaking existing reparametrizations. This has proven very useful to the authors as this enabled them to incrementally add the wealth of constraints now available, some of which are unique to *olex2.refine*, as discussed in Appendix *C* [App appc]. Furthermore, it enables third parties to develop their own constraints without the need for the involvement of the original authors beyond documenting how the reparametrization framework works.

A crystallographic refinement may involve many such reparametrizations. By piecing them all together, we obtain one reparametrization that expresses all refinable crystallographic parameters as a smaller vector of independent parameters that shall then be refined. Our framework safeguards that piecewise construction in several ways. First, at most one reparametrization may be applied to any given parameter. An attempt to add a reparametrization to a parameter that is already subject to one would be rejected by our framework as an error. Then if a cycle were found in the dependency graph, the framework would also reject the parametrization. This would happen when at least one parameter, through a series of reparametrization combinations, depends upon itself. Thus our framework safeguards against incorrect user inputs, and also against bugs in our own code that automatically builds constraints.

### 3.2. Constraint matrix   

There is a wealth of algorithms designed to minimize least squares, but crystallographic software has only implemented a few of them. The two most popular methods have historically been the full matrix^3^ (all small-molecule programs, including *Olex2*, as explained in Appendix *B* [App appb]) and conjugate gradient LS (CGLS, see *e.g.* Björck, 1996 ▶) which *SHELXL* offers as an option along with full matrix. The macromolecular community later introduced the limited-memory Broyden–Fletcher–Goldfarb–Shanno method (LBFGS, Nocedal, 1980 ▶), *phenix.refine* (Afonine *et al.*, 2012 ▶) and a sparse Gauss–Newton algorithm, *REFMAC* (Murshudov *et al.*, 2011 ▶, §4 and references therein). All methods mentioned above have in common the fact that they require only the computation of the value and the first-order derivatives of the calculated *F* or 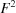, that we have denoted as 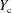 in this paper. Since no higher-order derivatives are used, the implementation of constrained least squares is greatly simplified.

Indeed, after transforming every constraint on the model into a reparametrization (as explained and exemplified in the previous section) and piecing all those reparametrizations together, we obtain a global reparametrization of the vector of crystallographic parameters 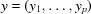 as a function of the vector 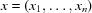 of the parameters that are actually refined. It should be noted that any component 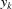 of *y* that is not reparametrized – *e.g.* a coordinate of the pivot atom on which another atom rides, and of course any parameter that is not involved in any constraint – is also a component 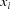 of *x*, *i.e.*

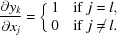



From the known analytical expression of 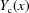, one computes the derivatives of 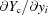 (the reader is referred to Appendix *A* [App appa] for a detailed presentation of those computations). The minimization algorithm then only needs the derivatives of 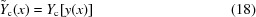
with respect to each 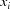, which are easily obtained by a simple application of the chain rule 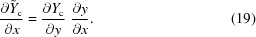
The matrix 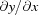 is known as the constraint matrix in crystallographic circles.^4^ In standard mathematical nomenclatures, it is called the Jacobian of the transformation 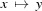 and we will therefore denote it *J*.

The computation of *J* takes advantage of the fact that it is a very sparse matrix. This stems from the fact that any given crystallographic parameter 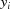 depends on very few refined parameters 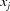, as amply illustrated in the previous section. We take advantage of this property to drastically reduce the memory cost of *J* and the cost of computing each of its non-zero coefficients. More precisely, both of these costs scale as the number of non-zero elements in *J* instead of 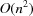 if *J* were treated as a dense matrix.

## 4. Restrained least-squares refinement   

In this section we will give an overview of the computations involved in restrained refinement. The mathematical formulae for each restraint objective and their derivatives can be found in Appendix *D* [App appd].

Since each restraint target 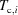 only depends on a very small subset of the 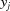 (*e.g.*, in the case of a bond length, only the six coordinates of the two bonded atoms would play a role), the matrix of derivatives with respect to the crystallographic parameters 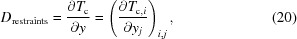
which is known as the design matrix for the restraints, is very sparse. We therefore use sparse-matrix techniques to efficiently store and perform computations with *D*, by only storing non-zero elements, and never performing any multiplication that involves an element of *D* known to be zero. We then introduce the matrix of derivatives with respect to the refined parameters, 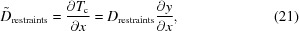
which is computed by forming the product of *D* and the constraint matrix. Thus the restraints are initially built up without any knowledge of the constraint matrix. This greatly simplifies their implementation and it simplifies their use in a refinement program that does not use constraints. This organization of the computation does not incur any inefficiency as the product (21) is a cheap operation since both matrices are sparse, and it therefore scales as the number of non-zero elements, which is typically much smaller than the number of parameters *n*.

The two terms in the normal equations (2) coming from the restraints then read as the matrix product and matrix-vector product, 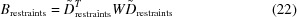


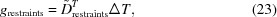
where 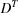 denotes the transpose of *D* and where *W* is the diagonal matrix featuring the restraint weights, and where 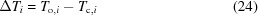
is the residual for the *i*th restraint. We again take advantage of the sparsity of 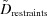 to efficiently implement those products.

It would be desirable to place the weights of the restraints on the same scale as the typical residual, such that a restraint will have a similar strength for the same weight in different structures. Rollett (1970 ▶) suggests the normalization factor 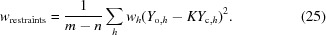
This is better known as the square of the *goodness of fit*, 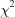. This normalizing factor also allows the restraints to have greater influence when the fit of the model to the data is poor (and the goodness of fit is greater than unity), whilst their influence lessens as the fit improves (Sheldrick, 1997 ▶).

### 4.1. Implementation   

The choice of the minimization algorithm has a significant impact on the organization of the computation of restraints. Indeed, a generic minimizer such as the LBFGS minimizer used in *phenix.refine* requires at each iteration only the function value *L* and the derivatives 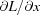. The derivatives 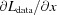 and 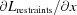 can be calculated separately before combining their sum to obtain 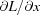. In contrast, for full-matrix least squares we need the matrices of partial derivatives 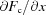 and 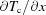. Therefore, depending on the optimization method used, we must be able to compute both 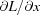 and 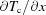 where by the chain rule 
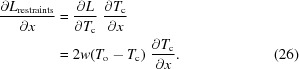



The restraints framework was designed in such a way that it could be easily extended by adding further restraints. Each restraint must provide the array of partial derivatives of the restraint with respect to the crystallographic parameters (one row of the matrix 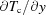), the restraint delta, 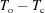, and the weight, *w*, of the restraint.

## 5. Twinning   

Like all other refinement programs, we have adopted the model of twins proposed by Jameson (1982 ▶) and Pratt *et al.* (1971 ▶). The sample is modelled as *d* domains, each sizeable enough a single crystal to give rise to observable Bragg peaks. If peaks from different domains fall very close in reciprocal space, the integration software analysing frames will be able to compute only the sum of the intensities of these superposed peaks. Data for the *r*th reflection will therefore consist of an intensity 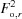 and of a list of 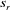 Miller indices 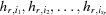 where 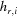 is the triplet of Miller indices of the Bragg peak originating from diffraction by domain *i*. The model of the structure then predicts an intensity 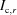 for 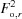 that reads 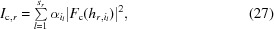
where 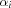 is the fraction of the sample volume occupied by twin domain *i*. The least squares to minimize are then, adapting equation (1) for a twinned structure, 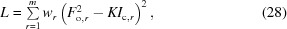
where the weight 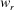 is usually 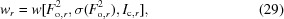
where *w* would be the same function discussed in the context of equation (1). The minimization of *L* with respect to the model parameters embodied in 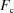 and with respect to the α’s is therefore subject to the constraint 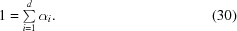



There is a special case that is common and therefore important, where there are exactly *d* superposed peaks for each reflection, *i.e.*, 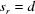 for every *r*, and where there is a 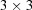 matrix *R*, the twin law, that generates the Miller indices for each domain 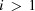 as 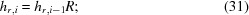
in this case, the input consists solely of a list of 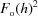 (as for an untwinned refinement but with 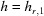) and of *R*. Such twins belong to the taxonomies pseudo-merohedral or merohedral. *olex2.refine* is able to refine a twinned structure input in this manner.

*SHELXL* users would handle the general case by using a reflection file in the HKLF5 format. Such a file is created by the integration software and this approach can deal with the most complex situations. By contrast, *CRYSTALS* always requires a list of twin laws.

*olex2.refine* can handle a more general case: when general and merohedral twinning are simultaneously present. For example, one may have four domains 1, 2, 3 and 4. Domains 1 and 3 are related by a twin law *R*, and so are domains 2 and 4, but the relative orientation of domains 1 and 2 does not correspond to any twin law. Thus the measured frames exhibit two lattices of Bragg peaks, with some overlaps, and the integration software will then output a list of (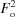, 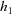), (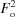, 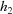) and (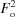, 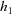, 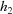). The refinement engine needs to expand this to a list of (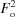, 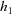, 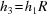), (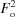, 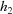, 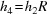) and (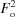, 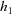, 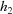, 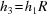, 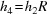). *olex2.refine* performs this task on the fly, while passing Miller triplets to the code computing structure factors and their derivatives.

The theory and phenomenology of twinning extends far beyond our exposition, but from the narrow point of view of refinement our presentation is sufficient, since, in this paper, we do not concern ourselves with the computation of the twin law or with the indexing of superposed lattices.

## 6. Standard uncertainties   

### 6.1. Variance matrix   

In this section, we will discuss the rigorous computation of s.u.’s of and correlation between the parameters of a constrained model. As pointed out in Appendix *B* [App appb] in the comment about equation (73), the variance matrix for the refined parameters *x* is 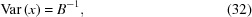
where 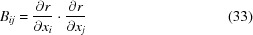
is the normal matrix for the constrained least-squares minimization. However, we are interested in the variance matrix 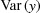 for the crystallographic parameters of the model, not in 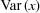. For small variations, we have the linear relation 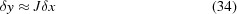
and therefore, using the well known heuristic definition of the variance matrix of *y* as the mean value of 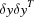 in the linear approximation around the minimum implicitly assumed throughout crystallographic refinement, 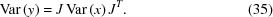



We would like to stress an important consequence of this formula: constrained parameters generally have non-zero s.u.’s. This is the case, for example, for the coordinates of riding atoms. For most constraints, the s.u.’s of hydrogen coordinates are equal to the s.u.’s of the atom they ride on but *e.g.* for a rotating –CH_3_, they differ because the s.u. of the azimuthal angle increases the s.u. of the hydrogen coordinates that come from riding only.

### 6.2. Derived parameters   

We will now discuss the s.u.’s of derived parameters. Such a parameter is a function *f* of a set of atomic parameters 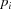 and its variance can be derived using the same heuristic as above in a linear approximation where 
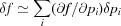
 by computing 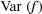 as the mean of 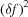. This leads to 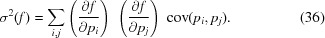
An early occurrence of this formula can be found in Sands (1966 ▶).

As a result of this formula and of the consequence of equation (35) stated after it, any bond length, any bond angle and any dihedral angle involving a riding atom has a non-zero s.u. unless that geometrical quantity is fixed by the constraint. For example, in the case of 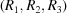
—C—H, for which the constraints do not fix the angles 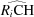, those angles have a non-zero s.u. This correct behaviour unfortunately triggers many alerts when a structure refined with *Olex2* is checked with *PLATON*.

*PLATON* plays a very important role in detecting common flaws in the crystallographic workflow. However, since *PLATON* does not have access either to the covariance matrix or the constraint matrix, it has to make guesses that result in estimation of e.s.d.’s that may be out by up to a factor 2. This is the key problem we encounter with the way *olex2.refine* reports e.s.d.’s. Specifically, most structures refined with *olex2.refine* fail *checkCIF* test PLAT732 (an alert C), for the reason explained in the documentation of this alert (in Note 2):^5^ *PLATON* computes the s.u. of the said angle using the s.u. of the atomic coordinates as if they were independent parameters since it does not have access to the variance–covariance matrix that describes their correlations. In this case, those correlations are very significant since the position of H completely depends on the position of *R*1, *R*2 and *R*3. Hence the s.u. of this angle as reported by *Olex2* differs from the *PLATON* estimate. Thus all failures of test PLAT732 for angles or distances involving constrained hydrogen atoms are spurious. As a result, if a referee were to require that all alert C’s are to be addressed in a CIF submitted for publication in *Acta Crystallographica*, we advise authors to explain away PLAT732 for riding hydrogen atoms by quoting Note 2 in the documentation for that test. *olex2.refine* will actually automatically prepare the CIF file with such explanations.

#### 6.2.1. Incorporating s.u.’s of unit-cell parameters   

Derived parameters such as bond lengths and angles are a function of both the least-squares atomic parameters and the unit-cell parameters. As such, the s.u. of a derived parameter is likewise a function of both the atomic and unit-cell parameters as well as their respective s.u.’s. If the s.u.’s in atomic parameters are considered to be totally uncorrelated with the s.u.’s in the cell parameters, *i.e.* their covariance is zero, then the s.u. in a derived parameter can be considered as comprising two independent sources of uncertainties: 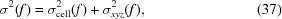
where 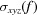 is the part coming from the uncertainties in the least-square estimates of the positional parameters, and 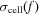 comes from the uncertainties in the unit-cell parameters, 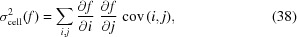
where 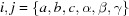.

This necessitates the calculation of the derivatives of the function with respect to the unit-cell parameters. In order to do so, it is easier to calculate separately the derivatives of the function with respect to the elements of the metrical matrix, and also the derivatives of the metrical matrix with respect to the cell parameters, and then to use the chain rule 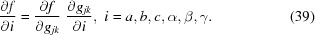
Indeed 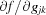 must be evaluated for every function, whereas 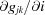 is constant for a given unit cell.

Now we consider the application of equation (36) to determine the s.u. in the length of the vector *u*, in fractional coordinates. The length, *D*, of the vector *u* is given by 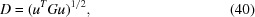
where *G* is the metrical matrix (see *e.g.* Giacovazzo *et al.*, 2011 ▶).

The derivatives of the distance, *D*, with respect to the elements of the metrical matrix, *G*, are given by 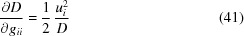
and (given the metrical matrix is symmetric) 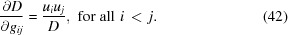



Similarly, for the angle between two vectors in fractional coordinates, *u* and *v*, where the angle is defined as 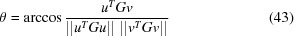
or 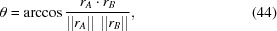
where 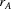 and 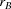 are the Cartesian equivalents of *u* and *v*, the derivative of the angle, θ, with respect to the elements of the metrical matrix, 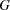, is given by 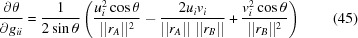
and 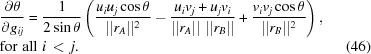



The derivatives of the metrical matrix with respect to the unit-cell parameters, 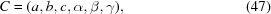
needed in order to apply equation (39) are given below: 
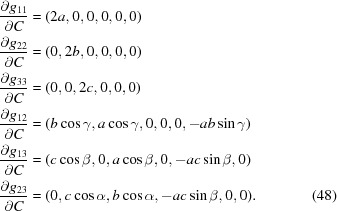



### 6.3. Symmetry   

The variance–covariance matrix that is obtained from the inversion of the least-squares normal matrix contains the variance and covariance of all the refined parameters. Frequently, it is necessary to compute functions that involve parameters that are related by some symmetry operator of the space group to the original parameters. Sands (1966 ▶) suggests that the symmetry should be applied to the variance–covariance matrix to obtain a new variance–covariance matrix for the symmetry-generated atoms. Alternatively, and it is this method that is used here, the original variance–covariance matrix can be used if the derivatives in equation (36) are mapped back to the original parameters.

Let the function *f* depend on the Cartesian site 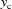 that is generated by the symmetry operator 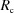 from the original Cartesian site 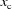, *i.e.*

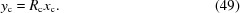
Then the gradient with respect to the original site can be obtained by 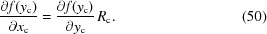



The variance–covariance matrix that is used in this case should be the one that is transformed to Cartesian coordinates. The variance–covariance matrix for Cartesian coordinates can be obtained from that for fractional coordinates by the transformation 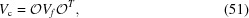
where the transformation matrix 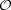 needed to transform the entire variance–covariance matrix in one operation would be block diagonal, with the 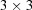 orthogonalization matrix *O* repeated at the appropriate positions along the diagonal. This transformation can be computed efficiently using sparse-matrix techniques.

## 7. Refinement results   

*olex2.refine* uses CIF (Hall *et al.*, 1991 ▶) as its main output. The CIF contains information regarding the space group, the data indicators such as merging indices, the refinement indicators such as the *R* factors, goodness of fit, residual electron density, refinement convergence indicators and tabulated structure information, including tables of atomic parameters, bonds and angles. *olex2.refine* produces a table describing the restraints using the CIF restraints dictionary. Moreover, the *olex2.refine* CIF always contains a verbal description of the refinement model – hydrogen-atom treatment, constraints, restraints and their targets. Optionally, the refinement model file (*SHELX* RES file) and the reflections can also be included in the final CIF for deposition. It should be noted that the constraints and restraints unique to *olex2.refine, i.e.* not featured by *SHELX*, are saved in REM sections (using an XML format). This has the advantage that they can be read back by *Olex2* while providing a ‘.res file’ that can also be refined with *SHELX*, albeit with potentially a different model. As part of this work a CIF-handling toolbox (Gildea *et al.*, 2011 ▶) was added to *cctbx*.

## 8. Conclusion   

We have presented herein the full mathematical derivations of the concepts used within *olex2.refine*. This new refinement engine is feature-wise on a par with the established software in the field such as *SHELXL* and *CRYSTALS*. It actually provides a richer wealth of constraints than those classic suites. *olex2.refine* is immediately useful to the practising crystallographer since it is presently available from the program *Olex2* by default. It can also be used independently as a library of components to write short scripts as well as more complex programs, dealing with any aspect of small-molecule refinement. Indeed, *olex2.refine* is largely based on the Small-Molecule Toolbox (*smtbx*) that is part of the Computational Crystallography Toolbox (*cctbx*), which is usable as a library for the Python programming language, thus providing great expressiveness, conciseness and ease of coding combined with an immense wealth of tools covering all of crystallography. We have explained herein how the *smtbx* added constrained least-squares refinement from scratch to the *cctbx* and how it added many restraints as well, thus opening new fields to the *cctbx*.

## Footnotes

1 EPSRC grant ‘Age Concern: Crystallographic Software for the Future’ (EP/C536274/1).

3 This method is also known as the Gauss–Newton algorithm.

4 The earliest appearance of that concept in crystallographic literature, though not by that name, brought to the attention of the authors is by Larson (1980 ▶).

5 This note seems to be a recent addition and the authors would like to thank Ton Spek for this helpful consideration.

## Appendix A Computation of structure factors and their gradient

The formulae discussed in this appendix have been known for nearly a century and have been implemented in all known refinement programs. However, it seems to the authors that, during the last decades, the computation for a given Miller index *h* of  and its gradient  with respect to crystallographic parameters has very rarely been presented in all the minute details necessary to implement those formulae in a program, justifying this appendix in our humble opinion. We will compare our algorithm to Rollett (1965 ▶) point by point as we present it, but first we would like to highlight a technical difference. Rollett computes the real and imaginary parts separately, whereas our computation is performed with complex numbers, the rationale being that the composition of the different terms of the structure factor can be performed with mere complex multiplications. For example, the incorporation of anomalous scattering in equation (53) corresponds to equations (20) and (21) in Rollett.

### A1. Structure factor of one atom

Since the structure factor of the entire unit cell is the sum over the contribution of each scatterer, we will focus on one such contribution only. We therefore consider a scatterer with fractional coordinates *x*, a thermal displacement tensor *U* in fractional coordinates and a chemical occupancy *s* (*i.e.* this occupancy does not take crystallographic symmetries into account). Its contribution  to the entire unit cell is obtained from its structure factor  by the sum

 denotes the subset of symmetry operators  of the rotational part *R* and translational part *t* that generate the orbit of the position *x* under the application of the entire set of symmetries in the space group of the structure, whereas  is the transform of  under the symmetry . For a spherical atomic model with an elastic scattering factor  that does not depend on the direction of *h* and an inelastic scattering factor  that does not depend on *h* (a property that holds for X-ray diffraction), *F* reads

We will consistently denote a triplet *h* of Miller indices as a row vector, whose transpose  is therefore a column vector, and a triplet *x* of fractional coordinates as a column vector. In this context,  denotes the Euclidean square of *h*, that therefore involves the reciprocal metric matrix  as .

If the thermal displacement is isotropic, the term  is replaced by  and it is then factored out of .

We are therefore left with computing the sum of the transforms of  under the symmetries in , the other terms forming a factor multiplying this sum. By the definition of a Fourier transform, that transform reads

Let us consider the case of non-primitive unit cells first. The sum can be partitioned as follows:

where  is the set of all centring translations, including zero, and  is the set of ‘primitive’ symmetries. Then with equation (54) we can factorize the sum as

The first term of the right-hand side does not depend upon the scatterer and it can therefore be precomputed. Commonly, Miller indices satisfy the centring conditions . In this case, this term is equal to the number of centring translations. However, this assumption can be invalid in the refinement of some pseudo-merohedral twins since Miller indices satisfying a centring condition may be transformed by the twin law in Miller indices that do not satisfy it.

It should be noted that Rollett already advocated skipping the computation of the terms in  corresponding to non-zero centring translations and then multiplying the result by 2. However, our framework can handle any group , not only those with  translations. It would work for tripled cells as well, for example.

Thus we are left with the computation of the second term of the right-hand side of equation (55). This is equivalent to treating the case of a primitive unit cell. Three cases are to be considered.

(i) The space group is non-centric. Then there is no further simplification.

(ii) The space group is centric but the inversion  may not be located at the origin. Then the sum over the symmetries may be split into the sum over the set  of proper symmetries and the sum over the set of improper symmetries. Since every improper symmetry may be written as  where  is a proper symmetry, we have

However, the product involving the inversion simplifies as  and therefore using equation (54)

but then from the very definition of  in equation (54)

and therefore

(iii) If , which holds true for every reflection if the space group is origin centric (), the previous case resolves to the real part of Σ only,

The total sum over symmetries is therefore real, and is its derivative, the former involving a cosine and the latter involving a sine for the derivatives with respect to *x*.

In order to avoid redundant computations, our code distinguishes these three cases. One piece of code handles the case of centrosymmetric space groups using only real numbers to compute Σ (an equivalent optimization was presented in Rollett). Another piece of code handles the other two cases, first computing Σ using complex number algebra and then using equation (57) if the space group is centric and  (an optimization not found in Rollett). Another optimization we applied is to precompute the terms *hR* and  appearing in equation (54) as well as the test for the condition  before the loop over all scatterers in the asymmetric unit (an optimization already advocated by Rollett). In all three cases, the computation of  and  is the most costly operation. This is mitigated by computing the structure factor and its derivatives together, as the cosine and sine then only need to be computed once for each reflection and scatterer. This optimization is the reason why we have written our own new code into the *smtbx* instead of reusing the original code in the *cctbx*. Indeed, in the latter, structure factors are computed separately from the derivatives, resulting in two separate loops over the reflections and the scatterers, and in sines and cosines being computed twice. This is rather well suited to the optimization algorithm used in *phenix.refine* (LBFGS) because it does not need the derivatives at every step. But it does not fit well with full-matrix least squares as is prevalent in small-molecule crystallography.

Our code also provides two options to compute the sines and cosines: either by using the trigonometric functions of the standard C++ library, or by using a *cctbx* function that interpolates between tabulated values of sines and cosines. The latter is much faster but less precise. *Olex2* uses the former as *CRYSTALS* and *SHELXL* use the standard FORTRAN sin and cos functions. It should be noted that Rollett used an approximation of trigonometric functions by Chebyshev polynomials, which used to be employed by *CRYSTALS*.

### A2. Derivatives of |*F*|^2^ and |*F*|

Having computed the unit-cell structure factor  and its derivatives, we need to compute the derivatives of  and perhaps as well  if performing a refinement against *F*. Since , where  denotes the complex conjugate of the complex number *z*, for any crystallographic parameter ξ,

but since the parameter ξ is always real in practice,

and therefore

where  denotes the real part. Then,

The *smtbx* provides code to compute derivatives for both of these observables. However, *Olex2* currently only offers the choice to refine against .

### A3. Extinction correction

*olex2.refine* models primary and secondary extinction with the same empirical correction used in *SHELXL*,

where the so-called extinction parameter *x* is added to the set of refined parameters. As noted in *SHELXL* documentation, it is close to the work of Becker & Coppens (1974 ▶) but not identical.

## Appendix B Minimization of least squares with an overall scale factor

In this appendix, we are concerned with the minimization of  as defined in equation (1). We will drop the label ‘data’ throughout this appendix for clarity. We will denote by  the values of those parameters at which  reaches the minimum we are interested in.

It is well known that the overall scale factor tends to be highly correlated with the thermal displacements. As a result, a starting value of *K* far from  tends to destabilize the fit and at the very least will increase the number of iterations necessary to converge to the minimum. It is, however, easy to compute a reasonable approximation of . Indeed, as a function of *K* only, keeping all the other parameters *x* fixed,  is a second-order polynomial. An analytical formula therefore exists for the value of *K* that minimizes that polynomial. Using the geometrical interpretation of least squares is the fastest manner to demonstrate this formula and leads to the most compact formula. We therefore introduce the scalar product of two sets of observables,

and the associated norm ,

as well as the residual vector

With those notations, , which reads

reaches a minimum at

while keeping *x* fixed, and the value at the minimum reads

We could then simply use  as a starting value for a combined refinement of *x* and *K*, *i.e.* the minimization of  with the independent vector  of parameters.

However, we chose to minimize  instead, which is a function of *x* only since  is completely determined by *x*. This is a special case of a technique called separable least squares (Nielsen, 2000 ▶, and references therein) that converges to the same minimum as previously, but with fewer iterations for the same cost per iteration. In order to carry out that minimization, we first need to compute the first-order derivatives,

where the chain rule would normally mandate a second term , but in this case it is zero because of the definition of . In this expression,

Thus, the first-order derivative is exactly the same as it would have been with an independent parameter *K*. We then need the second-order derivatives

where we have neglected the term  because, for a well behaved fit, the residual *r* and its curvature are small enough that this term can safely be neglected.

Let us now build the normal equations,

for the shift, *s*, of the parameter vector *x*. The matrix *B* is known as the normal matrix. Those equations are simply the Newton equations, featuring the Hessian matrix,  , computed using the approximation of equation (71) and the gradient *g* of . It should be noted that the solution, *s*, of equation (72) is then also the solution of the linear least-squares problem

The inverse of the normal matrix *B* when the least-squares minimum has been reached by  can therefore be viewed as the variance matrix of *x*, which should be contrasted to the more classic combined refinement of *x* and *K*, for which the variance matrix is the sub-matrix of the inverse of the normal matrix obtained by removing the column and the row corresponding to *K*.

Using the definition (65) of *r* and the expression (67) of , we have

and therefore the normal matrix *B* and the right-hand side of the normal equations *g* read

It should be noted that the first term of *B* would appear in exactly the same form if we had kept the scale factor *K* as an independent parameter. Moreover, terms very similar to the second and third terms of *B* would need to be computed in that case, as the normal matrix *B* would then have one more column and one more row which would feature those similar terms. Thus, the computation cost of our unorthodox method is the same as that of the more traditional refinement of the overall scale factor along with the other crystallographic parameters. In any case, for all methods based on the normal equations, the computation time is dominated by the first term of *B*, as it scales as , where *m* is the number of reflections and *n* the number of parameters.

It should also be noted that we never construct and store the whole of the so-called design matrix  that appears in those equations. Instead, we compute the vector of derivatives  for a few reflections *h* and then we immediately accumulate them into the normal matrix *B* and into the right-hand side , as opposed to constructing the whole design matrix and then forming the products  appearing in equation (76), for example. It used to be the only efficient way to proceed a few decades ago when least-squares refinement was being developed, but the fantastic increase of computer memory has made that point largely irrelevant.

The traditional argument is to show that the normal matrix is typically one to two orders of magnitude smaller than the design matrix. Indeed, the latter has  elements, whereas the former has approximately  elements for large values of *n*. Since in a typical small-molecule crystal structure determination the data-to-parameter ratio  is typically in the range 10–30, the normal matrix is therefore 20 to 60 times smaller than the design matrix. With the common use of constraints, particularly with respect to those on the parameters of hydrogen atoms, this contrast gets even more striking as *n* shall then be replaced with the number of parameters actually refined, which can be as small as  for typical organic structures. This results in another factor 2 in the comparison of the respective sizes of the design and of the normal matrix, and it makes the latter typically 40 to 120 times smaller than the former.

However, in the extreme case of a structure with about 1000 atoms in the asymmetric unit, the design matrix stored in double precision only requires 40 to 120 Mb of memory, assuming constraints reducing the number of refinable parameters by a factor 2, as opposed to around 2 Mb for the normal matrix. They would therefore both easily fit in the main memory of a modern PC which comes with many Gigabytes of RAM.

The counter-argument goes even further, as the normal matrix is actually not necessary either to solve the least-square problem or to compute estimated standard deviations for geometric quantities, which are necessary to publish a structure. Indeed, there are well known mathematical techniques based only on the design matrix to solve these two problems [*cf.* for example, Björck (1996 ▶), sections 2.8.3 for s.u. computations and 2.4.3 for the solution of the least squares].

However, the best of those design-matrix techniques, the QR decomposition, involves about twice as many floating-point operations as the method based on the normal equations. That is the actual motivation for our choosing the latter and therefore adhering to a long crystallographic tradition.

It should be noted that for singular or nearly singular problems, one could solve the LS problem by using singular-value filtering on the design matrix, which is the most robust technique. On the other hand, if one computes the normal matrix, therefore squaring the singularities, solving the LS problem with eigenvalue filtering is of little practical interest.

## Appendix C Constraints

*olex2.refine* provides constraints to influence the position(s) or the ADP(’s) of an atom or a group of atoms, as well as their occupancy factors. This section will enumerate the constraints and provide the details of their implementation.

### c1. Occupancy constraints

The *smtbx* provides a generic tool to express any scalar parameter *v* as an affine function of other scalar parameters ,

where the number of arguments *s* as well as the coefficients  and *b* can be freely chosen. The common case of two atoms whose occupancies *o* and  shall add up to 1 is easily handled by the case , as .

Higher values of *s* are useful to model a site partially occupied by different types of atoms when the requirement of full occupancy is complemented by other constraints. The example used in the *SHELXL* manual to illustrate the restraint command SUMP is such a case: a site is occupied by ions  and  with an average charge of +2, leading to the restrictions on their occupancies:

Instead of enforcing those relations as restraints, as advocated in the *SHELX* documentation, we can solve those equations to get the explicit dependencies

which can then readily be expressed as special cases of equation (77).

### c2. Shared site and shared *U* constraint

Two scatterers share the same site or the same ADP tensor. These constraints are pretty straightforward to implement within the *smtbx* framework. Values of dependent parameters and the corresponding rows of the Jacobian are set equal to the values for the reference atom.

### c3. Riding atoms/group *U* constraint

This constraint defined a group as riding on the given, pivot atom, *i.e.* the coordinates of the groups are given the same shifts as the pivot atom (*SHELXL* AFIX 3). Moreover the distances from the atom to the atoms of the group can be refined (*SHELXL* AFIX 4), which is applicable to groups like  for  where *X* is B, C, N *etc.* and *Y* is H, F, Cl *etc*. In general the transformation of the triplet of coordinates *r* is written down like this:

where *s* is the distance scaling component ( if not refined) and  is the pivot atom centre. In the case when *s* is refined, the corresponding derivative of the transformation is

whereas

### c4. Rotated *U* constraint

This constraint relates the ADPs of one atom to the ADPs of another atom as rotated by a fixed or refined angle around a given direction. The direction can be defined as a static vector, best line or best plane normal. Considering atoms *A* and *B*, the relation between their ADP tensor  and  reads

where *R* is the rotation matrix

with ,  and , and where the rotation direction is represented by a normal  and where α is the rotation angle around this direction. This transform is trivially rewritten as a linear transform of  into , whose matrix is therefore the Jacobian needed for the chain rule [equation (19)]. If the rotation angle is refined, then the derivative of this transformation by the angle is

where  is the derivative of *R* by α:

### c5. Pivoted rotation of a rigid body

This constraint is suitable for groups which can be rotated around a given direction (*SHELXL* AFIX 7). For groups  with  and *X* being B, C, N *etc.* and *Y* being H, F, Cl *etc.*, the distances from *X* to *Y* can also be refined (*SHELXL* AFIX 8). The coordinate transformation is

and its derivatives are

where  is the centre of the pivot atom, *s* is the distance scale component ( if not refined), *R* is the rotation matrix [equation (86)] and  is the derivative of the rotation matrix by the angle [equation (88)]. If neither the angle nor distances are refined, this constraint reduces to simple riding.

### c6. Free rotation of a rigid body

This constraint is the most generic case of the rigid-body refinement (*SHELXL* AFIX 6). In this case there are six refined parameters – the three Euler angles and three positional components; the ADPs are normally refined independently. The coordinate transformation in this case happens similarly to §C5; however a different rotation matrix is used:

and its derivatives by the angles are

For the special cases of spherical counter-ions or groups like Cp and Ph the size component of this constraint can also be refined (*SHELXL* AFIX 9).

### c7. Non-crystallographic symmetry constraint

This constraint is applicable to structures containing fragments which should be exactly superposable onto each other but which appear at different positions with different orientations. The same equations as for the free rotation of a rigid body described in §C6 are used in this case, but the coordinates of one of the group are refined along with the rotation angles and shift [*i.e.* equation (82) is used where all *r*’s are refined, contrary to the rigid-body case where those coordinates are fixed input, usually taken from a collection of known chemical groups]. This allows more observations to be used in the refinement of the atomic coordinates and ADPs.

## Appendix D Restraints and their derivatives

Possible restraints on the stereochemistry or geometry of atomic positions include restraints on bond distances, angles and dihedral angles, chiral volume and planarity. These restraints are used extensively in macromolecular crystallography, and hence were already implemented within the *cctbx* as part of the macromolecular refinement program *phenix.refine*. With the exception of the bond-distance restraint, these restraints were not able to accept symmetry-equivalent atoms. Since this is more frequently required in small-molecule crystallography, these restraints have now been extended to allow for symmetry. We have also implemented other restraints commonly used in small-molecule structure refinement, such as a bond similarity restraint, and restraints on anisotropic displacement parameters including restraints based on Hirshfeld’s ‘rigid-bond’ test (Hirshfeld, 1976 ▶), similarity restraints and isotropic ADP restraints.

### d1. Restraints involving symmetry

Given a restraint, , involving a site *x* which is outside the asymmetric unit and which is related to the site *y* within the asymmetric unit by some symmetry transformation , *i.e.* , the gradient is transformed as

### d2. Bond similarity restraint

The distances between two or more atom pairs are restrained to be equal by minimizing the weighted variance of the distances, where the least-squares residual, *L*, is defined as the population variance biased estimator

where  are the Euclidean distances between the atoms of each pair. The mean is defined as

where

and where  is therefore the weight for the *i*th pair.

The computation of the derivatives is easier with the alternate form of this variance,

The derivative of *L* with respect to a distance  is then

Then the derivatives of , where  and  are the Cartesian coordinates of the two atoms in that pair, are given by

and the same formula with . Therefore, using the chain rule, the derivatives of the residual with respect to  are

and the same formula with .

### d3. Planarity restraint

In this section, we will discuss a restraint enforcing a group of atom sites to be coplanar. We will first consider the case of four sites, with Cartesian coordinate vectors . They are coplanar if and only if the associated tetrahedron has a zero volume. This volume reads

and any circular permutation of , where  are any triplet of distinct edges of the tetrahedron. Choices that lead to elegant formulae are

and any circular permutations of .

Therefore a straightforward restraint enforcing coplanarity is the square of this volume, with a weight *w*,

Its derivative with respect to  is then easily obtained from equations (104) and (105):

The derivatives with respect to  are then obtained by circular permutations of , taking advantage of the behaviour of equations (104) and (105) with respect to permutations stated above.

We will now consider *p* sites with Cartesian coordinate vectors  that we want to restrain to be coplanar, with . We form the list  of tetrahedron  where . If the volume of the *i*th and (*i* + 1)th tetrahedron are, respectively, zero, then  are coplanar as the first four and the last four are, respectively, coplanar and those two quadruplets share a common triplet. Thus if all tetrahedra in  have a zero volume, then all sites are coplanar, which leads to building a restraint as the sum of the restraint for each tetrahedron,

This method is identical to the FLAT restraint in *SHELXL*, perhaps apart from differing implementation details.

The minimum number of degrees of freedom necessary to model *p* points in a plane is : 2*p* for the coordinates in the plane, plus two angles for the orientation of the plane, plus one for the distance of the origin to the plane. Since this number of degrees of freedom is exactly the unconstrained and unrestrained number of degrees of freedom 3*p* minus the number of restraints in the sum of equation (108), this restraint  is therefore optimal.

### d4. Restraints on atomic displacement parameters

We will discuss three types of restraints on anisotropic displacement parameters (Rollett, 1970 ▶), similar to restraints implemented in *SHELXL* and *REFMAC* (Murshudov *et al.*, 1999 ▶).

#### d4.1. Rigid-bond restraint

In a ‘rigid-bond’ restraint the components of the anisotropic displacement parameters of two atoms in the direction of the vector connecting those two atoms are restrained to be equal. This corresponds to Hirshfeld’s ‘rigid-bond’ test (Hirshfeld, 1976 ▶) for testing whether anisotropic displacement parameters are physically reasonable (Sheldrick, 1997 ▶) and is in general appropriate for bonded and 1,3-separated pairs of atoms and should hold true for most covalently bonded systems.

Since the mean-square displacement of an atom of thermal displacement tensor *U* along a normalized vector *u* is , the restraint residual for two atoms *A* and *B* at respective positions  and  and with respective thermal displacement tensors  and  reads

where

and the restraint term is then

Those formulae are valid whether using Cartesian or fractional coordinates, providing the adapted formula is used to compute , *i.e.*

in Cartesian coordinates and

in fractional coordinates, where *G* is the metric matrix.

The computation of the derivatives of *L* with respect to the components of  and  proceeds in two steps. First, since , where *U* is either  or ,

The chain rule then gives, where *U* is either  or ,

The derivatives of  with respect to the positions  and  are ignored.

#### d4.2. ADP similarity restraint

The anisotropic displacement parameters of two atoms are restrained to have the same  components. Since this is only a rough approximation to reality, this restraint should be given a smaller weight in the least-squares minimization than for a rigid-bond restraint and is suitable for use in larger structures with a poor data-to-parameter ratio. Applied correctly, this restraint permits a gradual increase and change in direction of the anisotropic displacement parameters along a side chain. This is equivalent to a *SHELXL* SIMU restraint (Sheldrick, 1997 ▶). The weighted least-squares residual is defined as

which, denoting  the matrix of deltas, is the trace of , making it clear that it is invariant under any rotation *R*, since it transforms  into .

Since  is symmetric, *i.e.* , equation (117) can be rewritten as

Therefore the gradients of the residual with respect to diagonal elements are then

whereas the gradients with respect to the off-diagonal elements are

and then the same equation with .

#### d4.3. Isotropic ADP restraint

Here we minimize the difference between the ADP’s , and the isotropic equivalent, defined in Cartesian coordinates by

and

Again, this is an approximate restraint and as such should have a comparatively small weight. A common use for this restraint would be for solvent water, where the two restraints discussed previously would be inappropriate (Sheldrick, 1997 ▶). As in equation (117), we define a restraint term invariant under rotations, as , or explicitly,

which simplifies in Cartesian coordinates into

By mere inspection, we can see that the derivatives of the residual with respect to the off-diagonal elements are in Cartesian coordinates:

#### d4.4. ADP *U*_eq_ similarity

The ADP of two atoms *A* and *B* may be restrained to have the same . The restraint term for atom *A* reads

or in Cartesian coordinates

This restraint is therefore invariant under rotation.

The derivatives with respect to the diagonal elements of  are then

and the same formula with .

#### d4.5. Fixed ADP *U*_eq_

When the  of an atom is restrained to a fixed value, *P*, the residual for this restraint is

and the derivatives of the residual with respect to diagonal values of *U* are

#### d4.6. ADP volume similarity

The volumes of the thermal ellipsoids of two atoms *A* and *B* may be restrained to be equal. The restraint term for atom *A* reads

The harmonic anisotropic displacement of an atom is described by a multivariate normal distribution of covariant matrix *U* (see *e.g.* Trueblood *et al.*, 1996 ▶), which is the anisotropic displacement tensor of that atom. Therefore, the probability distribution function is

The surface of constant probability *p* is therefore an ellipsoid of equation

where

In order to arrive at a simple formula, we fix the probability *p* such that the denominator is equal to 1, *i.e.*

The volume of an ellipsoid with such an equation is given by , *i.e.* with the chosen value of *p*,

whose derivatives read

We would like to emphasize once more that our choice of ellipsoid whose volume we wish to restrain lacks physical or mathematical motivations. It is only driven by simplicity. It is interesting to contrast the thermal ellipsoid volume restraint with the  similarity restraint. Indeed, the former restrains the sum of the eigenvalues of *U* whereas the latter restrains the product of those eigenvalues. They are therefore complementary.
